# Oral feeding for infants and children receiving nasal continuous positive airway pressure and high flow nasal cannula: a systematic review

**DOI:** 10.1186/s12887-021-02531-4

**Published:** 2021-02-17

**Authors:** Angie Canning, Sally Clarke, Sarah Thorning, Manbir Chauhan, Kelly A Weir

**Affiliations:** 1 Speech Pathology, Gold Coast University Hospital, Gold Coast Health , Gold Coast, Australia; 2 Queensland Children’s Hospital, Children’s Health Queensland, Brisbane, Australia; 3 Library Services, Gold Coast University Hospital, Gold Coast Health, Gold Cost, Australia; 4 Newborn Care Unit, Gold Coast University Hospital, Gold Coast Health, Gold Coast, Australia; 5 Allied Health Sciences & Menzies Health Institute Queensland Griffith University, Gold Coast, Australia; 6 Allied Health Research Gold Coast Health, Gold Coast, Australia

**Keywords:** Oral feeding, nCPAP, HFNC, Pediatric, Swallowing

## Abstract

**Background:**

The aim of this systematic review was to determine whether introduction of oral feeding for infants and children receiving nasal continuous positive airway pressure (nCPAP) or high flow nasal cannula (HFNC) respiratory support facilitates achievement of full oral feeding without adverse effects, compared to no oral feeding (NPO; nil per oral) on CPAP or HFNC.

**Methods:**

A protocol was lodged with the PROSPERO International Prospective Register of Systematic Reviews. We searched Medline, Embase, CINAHL, CENTRAL and AustHealth from database inception to 10th June 2020. Study population included children (preterm to < 18 years) on nCPAP or HFNC who were orally feeding. Primary outcomes included full or partial oral feeding and oropharyngeal aspiration. Secondary outcomes examined adverse events including clinical signs of aspiration, aspiration pneumonia and deterioration in respiratory status.

**Results:**

The search retrieved 1684 studies following duplicate removal. Title and abstract screening identified 70 studies for full text screening and of these, 16 were included in the review for data extraction. Methods of non-invasive ventilation (NIV) included nCPAP (*n* = 6), nCPAP and HFNC (*n* = 5) and HFNC (*n* = 5). A metanalysis was not possible as respiratory modes and cohorts were not comparable. Eleven studies reported on adverse events. Oral feeding safety was predominantly based on retrospective data from chart entries and clinical signs, with only one study using an instrumental swallow evaluation (VFSS) to determine aspiration status.

**Conclusions:**

Findings are insufficient to conclude whether commencing oral feeding whilst on nCPAP or HFNC facilitates transition to full oral feeding without adverse effects, including oropharyngeal aspiration. Further research is required to determine the safety and efficacy of oral feeding on CPAP and HFNC for infants and children.

**Trial registration:**

PROSPERO registration number: CRD42016039325.

## Background

The use of non-invasive ventilation (NIV), including nasal continuous positive airway pressure (nCPAP) and high flow nasal cannula (HFNC), has increased over the last two decades as primary or step-down respiratory therapies for infants and children with acute and chronic respiratory conditions [1–3]. They provide support for infants in neonatal intensive care units (NICU) with respiratory distress syndrome and bronchopulmonary dysplasia (BPD)/chronic neonatal lung disease (CNLD) [1, 4], and children in pediatric intensive care units (PICU) for treatment of acute illnesses such as bronchiolitis and pneumonia; and can reduce the need for invasive ventilation [5, 6]. However, the impacts of nCPAP and HFNC on oral feeding and swallowing are unknown [5, 7].

Historically, infants and children receiving nCPAP and HFNC were kept nil per oral and received tube feedings only, due to concerns regarding impacts on swallow safety (considered to be at a higher risk of oropharyngeal aspiration: fluid/food entering the airways below the level of the vocal folds) and cardiorespiratory stability [8–11]. HFNC increases pharyngeal pressures, which may affect laryngeal closure, pharyngeal sensory responses, and in turn, airway protection mechanisms [7, 11–14]. nCPAP is known to impact the timing and frequency of the swallow reflex in adults [15]. Thus, potential aspiration during oral feeding whilst receiving nCPAP and HFNC could increase the duration of respiratory support requirement, increase hospital length of stay and negatively impact feeding and respiratory outcomes.

As non-invasive respiratory therapies provide access to the mouth, oral feeding for infants and children receiving nCPAP and HFNC is increasingly being provided. In the preterm population the requirement for nCPAP or HFNC may coincide with infants’ developmental readiness for oral feeding. Therefore oral feeding experiences are provided in an attempt to support neurodevelopmental outcomes, facilitate transition to full oral feeding and reduce length of stay [8, 10, 16, 17]. For infants and children with acute respiratory illness, poor nutrition is thought to increase length of PICU stay, therefore oral feeding may be provided to optimise nutrition, reduce length of stay and for comfort [9, 13].

A recent survey of practice of NICUs and PICUs in Australian and New Zealand reported that most units surveyed do feed on NIV, more frequently on HFNC, and with use of strategies including monitoring stability and reducing pressure/flow rate during oral feeding. The primary reason for not orally feeding on NIV was that the aspiration risk is unclear The survey reported high variability in feeding practices, differing clinical opinion and a lack of evidence-based clinical guidelines regarding oral feeding for this cohort [18]. Therefore this systematic review aimed to determine if oral feeding for infants and children receiving nCPAP and HFNC facilitates full oral feeding without adverse effects (including oropharyngeal aspiration).

## Methods

The study protocol was conducted in accordance with the Preferred Reporting Items for Systematic Reviews and Meta-Analyses (PRISMA) Statement and lodged with the PROPERO International Prospective Register of Systematic Reviews (CRD42016039325) [19].

A comprehensive search was conducted by a medical librarian (ST) using the following databases: Medline (Ovid), Embase (Elsevier), CINAHL (Ebsco), The Cochrane Central Register of Controlled Trials (CENTRAL) and AustHealth (Informit) from database inception to 10th June 2020. Manual searching of reference lists of studies retrieved for data extraction was undertaken. There was no restriction on publication date or language. The search strategy included the following keywords or Medical Subject Headings (MeSH) terms: 1) suck or feed or oral or bottle or breast or nipple or infant feeding; and; 2) high flow and nasal cannula or nasal prong or oxygen; cpap or ncpap or bcpap or peep or positive end expiratory pressure or continuous positive airway pressure or positive end expiratory pressure. The full search strategy is documented in the PROSPERO protocol [19].

Included studies met the following criteria: 1) pediatric population (birth to < 18 years); 2) participants received oral feeding/nutritive swallowing (i.e., breast, bottle feeding, cup drinking, solids intake); 3) participants received nCPAP or HFNC therapy at the time of oral feeding/nutritive swallowing; 4) study types included randomised control trials, control trials, cohort studies, case series and case reports. Grey literature was not included.

The criteria were kept deliberately broad to encompass children at different ages and stages of their feeding development, as this reflects the children who we see clinically at our tertiary institution. Oral feeding was defined as any amount of fluid/food taken by mouth. Studies were excluded if they were adult populations (≥18 years of age); received only low flow nasal cannula (LFNC) support or invasive ventilation; or participants were nil per oral (parenteral/tube feeding only).

Three primary outcomes were established:

1. Full oral feeding (receiving all nutrition and hydration by mouth and no longer receiving tube/parenteral feeding)
2. Partial oral feeding (defined as ‘oral feeding with supplemental tube/parental feeding’ or ‘oral feeding without full oral feeding’ reported as an outcome)
3. Oropharyngeal aspiration, as observed on instrumental assessment (videofluoroscopic swallow study or fiberoptic-endoscopic evaluation of the swallow)

Secondary outcomes examined adverse effects including:

1. Clinical signs of oropharyngeal aspiration
2. Aspiration pneumonia or use of antibiotics for clinically suspected aspiration pneumonia
3. Deterioration in respiratory status or respiratory distress (increased work of breathing/oxygen requirements, oxygen desaturations, chest x-ray findings)
4. Oral aversion/feeding refusal
5. Behavioural responses (e.g. gagging/disengagement/refusal cues)
6. Gastro-oesophageal reflux
7. Death

Initial screening of the title, abstract and keywords, and full text reviews were performed by two authors (AC,SC) according to the inclusion and exclusion criteria using Covidence [20]. Disagreements were resolved by consulting a third author (KW) and consensus reached. Two review authors (AC,KW) independently performed data extraction and discrepancies resolved through discussion or with a third author (SC).

Quality assessment of the included studies was undertaken independently by two reviewers (AC,KW) and disagreements resolved through discussion. Risk of bias was determined using the Cochrane Risk of Bias in Non-randomized Studies (ROBINS-1) [21] and The Cochrane Risk of Bias (ROB-2) [22] tools. Publication bias was not assessed due to the small number of trials.

## Results

**Fig. 1:**
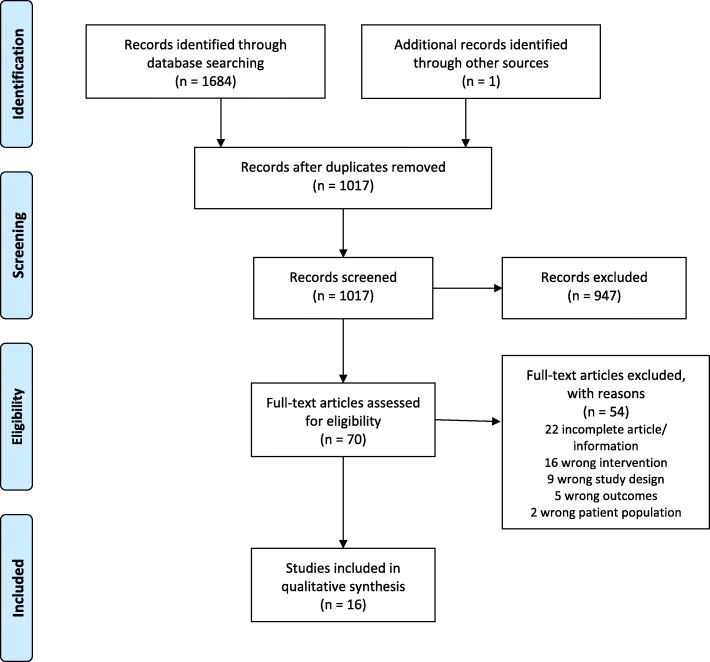
PRISMA flow chart- included and excluded studies

Database searching retrieved 1684 records after duplicates were removed. One further record was added through reference list searching. Title and abstract screening identified 70 studies for full text screening. Full text screening identified 16 studies that met eligibility criteria for inclusion in the review and 54 studies were excluded. See Fig. 1 for PRISMA flow diagram.

### Study characteristics

See Table 1 for a summary of study characteristics.

**Table 1:** Study characteristics

Author, year	Study design and setting	Participants (sample size, age and condition)	Type of respiratory support, flow/pressure	Details of oral feeding	Main outcomes
**Bapat 2019** [23]	Quality improvement project (non-contemporary cohort comparison study); NICU	279 infants < 32 + 6 weeks GA (198 had BPD); baseline group 92 infants (63 had BPD); SIMPLE group 187 infants (135 had BPD)	CPAP (H2O not reported)	Oral feeding on CPAP; Guideline for feeding strategies on respiratory support; once a day oral feeding by occupational therapist, intensive cautious early feeding opportunities.	Days to full enteral feeding; days to first oral feeding; days to full oral feeding; ventilation duration; growth milestones; discharge milestones including LOS
**Dalgleish 2016** [10]	Quality improvement project (non-contemporary cohort comparison study); NICU	196 infants born < 32 weeks with respiratory morbidity	CPAP (cmH2O not reported) HFNC> 1.5 L/min	Cohort 1: No oral feeding on NIV = 91; Cohort 2: Oral feeding on NIV = 105; Oral feeding on nCPAP as per novel algorithm ‘Eating in SINC: Safe Individualised Nipple-Feeding Competence’	GA at first oral feed; days of respiratory support; respiratory support at first NF; LOS; safety
**Dumpa 2020** [24]	Retrospective cohort study; NICU	99 infants < 32 weeks GA	CPAP 5-8cmH2O	Group 1 (oral feeding commenced on CPAP) = 39; Group 2 (oral feeding commenced when off CPAP); objective oral feeding assessment developed by NICU staff.	Duration to achieve full oral feeding; LOS; respiratory morbidities
**Ferrara 2017** [14]	Prospective cohort study; NICU	7 infants with a PMA > 34 weeks 6 preterm, 1 term (34.1–43.2 weeks CGA)	CPAP 5cmH2O LFNC 1 L/min	Oral feeding on CPAP; Infant swaddled positioned in a sitting position in a tumbleform infant seat, bottle offered for 90 s by a single feeding and swallowing specialize.	Incidence of mild and deep laryngeal penetration, aspiration and nasopharyngeal reflux on VFSS
**Glackin 2017** [25]	Randomised control trial; NICU	44 infants born before 30 weeks nCPAP = 22; HFNC = 22	nCPAP (cmH2O not reported, stated ‘current setting’); HFNC commencing at 7 L/min	Oral feeding on CPAP and HFNC; Oral feeds offered in both groups at least once every 72 h and additional feeds offered when infants demonstrated feeding cues.	Duration to first oral feed; duration to full oral feeds; duration of resp. support; CNLD; LOS; episodes of apnoea
**Hanin 2015** [26]	Retrospective cohort study; NICU	53 infants with BPD 37-42wks PMA;	nCPAP 6-8cmH2O	Orally fed on nCPAP = 26; Gavage fed on nCPAP =27; All oral feedings were done by a trained neonatal OT; clinical assessment completed prior to initiation of feeding therapy; based of SOFFI method; oral feeding session no more than 30mins, one session per day, 3–5 times per week.	Duration to full oral feeds; LOS; duration of nCPAP; safety metrics; readmission rate
**Jadcherla 2016** [27]	Prospective case control study; NICU	38 infants with BPD 28 + 0.7wks GA; 39-43wks CGA at evaluation; nCPAP = 9; NC = 19; RA = 10	nCPAP 6-8 cm H2O; NC 0.1–2.0 L/min	Graded sterile water infusions via syringe of 0.1, 0.3 and 0.5 mL to the pharynx for infnats on CPAP.	Effects of pharyngeal stimulation on the initial and terminal pharyngoesophageal and respiratory responses
**La Tuga 2019**	Retrospective case control study; NICU	243 infants < 32 weeks GA who required CPAP at 32 weeks PCA	CPAP (cmH2O not reported)	No CPAP first oral feed GA 27 (24–32) wks; CPAP first oral feed GA 26 (23–32) wks 31% (*n* = 76) received first oral feed on CPAP; Oral feeding defined as any feeding taken by mouth > 5 mL	Length of stay; duration of resp. support; age at first oral feed; age at full oral feeds; duration to full oral feed; aspiration pneumonia
**Leder 2015**	Prospective cohort study; NICU & adult ICU	100 participants: 50 neonates (CGA range 33w7d-49w3d) & 50 adults	HFO2-NC 2-3 L/min	Oral feeding on HFNC. 17 neonates had oral feeding. Decisions to initiate oral feeding made jointly by neonatology and nursing using criteria.	Successful initiation of oral feeding; age at initiation of oral feeds
**Leibel 2020** [33]	Randomised control pilot study; NICU	25 infants born < 28 weeks GA, 34 weeks PMA, requiring CPAP or HFNC’; CPAP *n* = 12; HHHFNC *n* = 13	CPAP >5cmH2O; HHHFNC > 5 L/min	Infants on CPAP were placed on LFNC (up to 2 L/min) for oral feeding, infants on HHFNC had flow reduced to 2 L/min for oral feeding	Days to full oral feed; weight gain; feeding type; feeding intolerance; NIV support at end of trial; incidence of CLD; PMA at conclusion of trial
**Leroue 2017** [28]	Retrospective cohort study; PICU	562 children older than 30 days to > 10 years (median age 2 yrs) requiring NIPPV, majority had a primary diagnosis of bronchiolitis or viral pneumonia	NIPPV = HHFNC, CPAP, BiPAP, AVAPS; CPAP or bilevel support 6-8cmH2O; HHFNC (flow rate/s not reported)	Oral feeding on NIPPV. 305 (54%) had oral intake.	Early EN; time to goal EN rate; adequacy of EN; frequency of EN interruptions > 6 h; AEs
**Shadman 2019** [29]	Retrospective cohort study; intensive and general care units, children’s hospital	123 children aged 1 to 24 months with bronchiolitis treated with HFNC	HFNC (flow rate/s not reported)	Oral feeding on HFNC. 78 (63%) were fed: 50 (41%) were exclusively orally fed and 28 (23%) had mixed oral and tube feeding.	Time to discharge after HFNC cessation; aspiration; intubation after HFNC; seven-day readmission
**Shetty 2016** [8]	Retrospective cohort comparison study; NICU	116 infants with BPD (24-32wks GA); nCPAP =72; nCPAP/HHFNC =44	CPAP 4-6cmH2O; HHFNC 2-8 L/min	Oral feeding on HFNC (no oral feeding on CPAP); Infants on HFNC were referred to SLT service from 34 weeks GA to assess readiness to cope with oral feeding.	Age at first oral feed; age at full oral feeds; duration and type of resp. support; LOS
**Shimizu 2019** [30]	Retrospective case control study; NICU	45 infants (< 34 weeks PMA; GA 23.1–39.6 weeks GA) with very low birth weight and chronic lung disease	HFNC 2 L/kg/min	Oral feeding on HFNC *n* = 11 (GA 27.4; 23.1–32.0 weeks); oral feeding without HFNC *n* = 34 (31.2; 23.7–39.6 weeks); Oral feedings offered to infants with stable breathing after 34 weeks PMA, after oral feeding skill evaluation by physical therapists.	Duration to first oral feed; duration to full oral feeds; clinically significant aspiration pneumonia
**Slain 2017** [9]	Retrospective cohort study; PICU	70 children < 24 months (median age of 5 months) with bronchiolitis	HFNC 2-4 L/min; 5-6 L/min; > 7 L/min	Oral feeding on HFNC; 89% fed orally.	Incidence of feeding-related AEs; LOS; duration of HFNC
**Sochet 2017** [31]	Prospective cohort study; PICU	132 children (1 month to 2 yrs) with bronchiolitis	HFNC 4-13 L/min (0.3–1.9 L/kg/min)	Oral feeding on HFNC; 97% fed orally.	Incidence of aspiration-related respiratory failure

*nCPAP* nasal continuous positive airway pressure, *HFNC/HHFNC* (humidified) high flow nasal cannula, *HFO2-NC* high flow oxygen nasal cannula, *NC* nasal cannula, *NIPPV* nasal intermittent positive pressure ventilation, *BiPAP* bilevel positive airway pressure, *AVAPS* average volume assured pressure support, *RA* room air, *GA* gestational age, *CGA* corrected gestational age, *PMA* postmenstrual age, *PCA* post-conceptual age, *BPD* bronchopulmonary dysplasia, *AEs* adverse events, *LOS* length of stay, *EN* enteral nutrition, *VFSS* videofluoroscopic swallow study, *SOFFI* Supporting Oral Feeding for Fragile Infants [32]

Five retrospective cohort studies [9, 24, 26, 28, 29], three prospective cohort studies [14, 16, 31], two randomised control studies [25, 33], two retrospective case control studies [30, 34], two quality improvement projects (non-contemporary cohort comparison studies) [10, 23], one prospective case control study [27] and one retrospective cohort comparison study [8] were included in this review.

Study sample sizes ranged from seven to 562 participants. In one study [16] involving both adult and neonatal patients, only the neonatal data was included in the review. Twelve studies included participants from NICUs with five studies [8, 23, 26, 27, 30] including infants with a diagnosis of CNLD/BPD, whilst seven studies [10, 14, 16, 24, 25, 33, 34] included patients with respiratory morbidity requiring NIV with no further diagnostic specification. Three studies [9, 28, 31] were from PICU settings and one study was from both intensive and general care units in a children’s hospital [29]. Three of these studies included children aged 0 to 24 months with bronchiolitis [9, 29, 31] and one study (*n* = 562) included children aged 30 days to 10 years with a range of diagnoses including bronchiolitis (48%), viral pneumonia (16%) and status asthmaticus (18%) [28].

Six studies [14, 23, 24, 26, 34] included participants receiving nCPAP only, with three studies reporting on nCPAP pressures. In five studies [9, 16, 29–31] participants received HFNC only, with flow rates reported in four studies. Five studies [8, 10, 25, 28, 33] included participants receiving nCPAP and HFNC.

### Main outcomes

Main outcomes included age at/duration to first oral feed, age at/duration to full oral feeds, adequacy and frequency of enteral nutrition, adverse events, duration of respiratory support, length of stay, number of participants discharged with tube feeding, type of feeding at discharge and readmission rate.

### Quality of individual studies

**Fig. 2:**
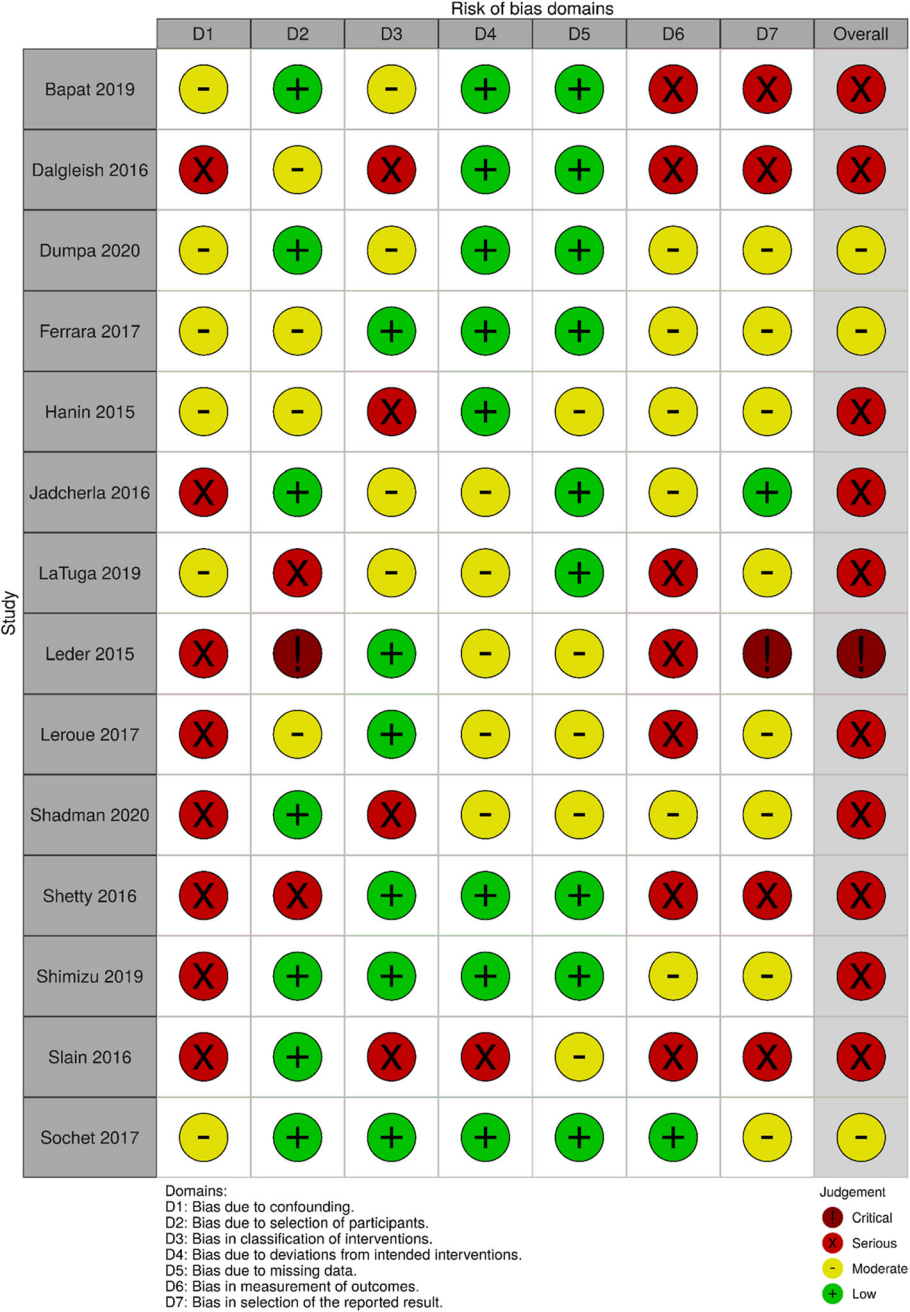
Risk of bias plot for non-RCT studies [35]

**Fig. 3:**
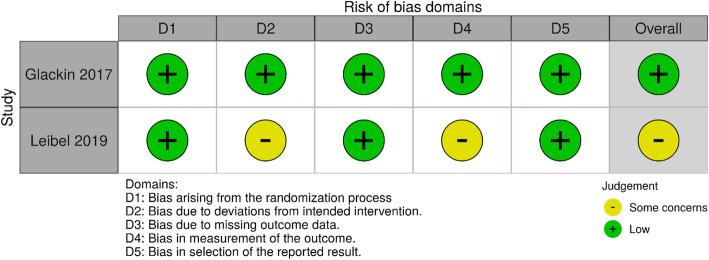
Risk of bias plot for RCT studies [35]

For the 14 included non-RCTs, three studies were judged to have a moderate risk, 10 studies a serious risk and one study a critical risk of bias. Due to the nature of the research designs, no studies were judged to have a low risk of bias. Investigators were not blinded and allocation to intervention was sometimes based on physician judgment. One included RCT had an overall judgment of ‘some concerns’ due to risk of bias arising from deviations from the intended interventions, and the other included RCT was judged to have a low risk of bias. Refer to Figs. 2 and 3 for risk of bias summaries.

### Results of individual studies

The following information was extracted from the included studies against the outcome measures established for this review.

#### Analysis of primary outcomes

See Table 2 for a summary of primary outcomes.

1. Full oral feeding

**Table 2:** Summary of primary outcomes (PO)

Study, year	Respiratory support	PO1: Full oral feeding	PO2: Partial oral feeding (including initiation of oral feeding)	PO3: OPA- instrumental assessment	Conclusion/s
**Bapat 2019** [23]	CPAP (cmH2O not specified)	**Full oral feeds** were achieved significantly earlier by infants with mild to moderate BPD (but not for severe BPD) in the SIMPLE feeding program. Baseline group median 84 DOL (range 90 + 32DOL), SIMPLE group median 81 DOL (range 85 + 36DOL)	**First oral feed** milestone achieved at an earlier age for the SIMPLE feeding group for all 3 severity categories (mild, mod, severe BPD). Baseline group median 72 DOL, SIMPLE group median 64 DOL.	No	Intensive cautious early feeding opportunities may be helpful in modifying the aerodigestive outcomes among BPD patients. The SIMPLE feeding strategy **advances maturation and acquisition** of feeding milestones irrespective of the severity of BPD and impacts LOS.
**Dalgleish 2016** [10]	**CPAP** (pressure/s not reported) **HFNC** > 1.5 L/min	Not reported	Age at **first oral feed** GA mean 32 weeks, 4 days; 65 (61.9%) of the 105 participants were no longer receiving nCPAP when oral feeds were initiated.	No	Project suggests the consistent approach for NF may be safe in the short-term, however is a pilot study with plans for further evaluation of safety and efficacy of the SINC strategy
**Dumpa 2020** [24]	Orally fed while on **nCPAP** 5-8cmH2O (group 1) vs oral feeding after ceasing nCPAP (group 2)	Group 1 took longer to achieve **full oral feeding** (median 16 days) vs group 2 (median 10 days) vs group 3 (PMA > 34 weeks, off nCPAP, positive oral feeding cues) (median 10 days). However PMA at full oral feeding reached was not significantly different between the groups.	Infants in group 1 had an earlier **initiation of oral feeds** (median PMA 35.2 weeks), as expected, compared with group 2 (median PMA 35.8 weeks) and group 3 (median PMA 35.9 weeks).	No	Delaying oral feeding until ceasing nCPAP did not result in feeding-related morbidities. Caution recommended when initiating oral feedings in preterm infants on nCPAP without evaluating the safety of the infants and their readiness for oral feeding.
**Ferrara 2017** [14]	**CPAP** 5cmH2O vs **LFNC** 1 L/min	Not reported	Tolerating at least 50% of TFI orally	Yes	Oral feeding while on-nCPAP significantly increases the risk of laryngeal penetration and tracheal aspiration events. Recommend caution when initiating oral feedings on nCPAP.
**Glackin 2017** [25]	**nCPAP** (pressure/s not reported) vs **HFNC** commencing at 7 L/min	Number of days to achieve **full oral feeding** was found to not be significantly different between the nCPAP and HFNC cohorts (HFNC 36.5 days + 18.2; nCPAP 34.1 days + 11.2; *p* = 0.61).	**First oral feed** (days from enrolment at 32 weeks CGA) for infants receiving nCPAP (9.3 + 6.5 days) and HFNC (10.9 + 4.8 days), *p* = 0.37. 6 infants in nCPAP group (n = 22) and 1 in HFNC group (*n* = 22) were off respiratory support when the first oral feed was provided.	No	Preterm infants treated with HFNC **did not achieve full oral feeding more quickly** than infants treated with nCPAP.
**Hanin 2015** [26]	**nCPAP**-oral (6-8cmH2O) vs nCPAP-gavage	nCPAP-oral fed group achieved **full oral feeding** 17 days earlier (median) compared with the infants on nCPAP that were not orally fed and gavage/tube fed only (nCPAP-oral 120.5 DOL, 41.6 weeks PMA; nCPAP-gavage 137 DOL; 45.5 weeks PMA; *p* > 0.05).	Not reported	No	Controlled introduction of oral feedings in infants with BPD during nCPAP is safe and **may accelerate** the acquisition of oral feeding milestones.
**Jadcherla 2016** [27]	**nCPAP** (6-8 cm H2O) **NC** (0.1–2.0 L/min) Room air	Not reported	Graded sterile water infusions via syringe of 0.1, 0.3 and 0.5 mL to the pharynx.	No	The current study lends support to provide mechanistic basis and rationale for supporting “controlled and regulated” oral feeding during nCPAP or HFNC.
**La Tuga 2019**	**CPAP** (pressure/s not reported) vs no CPAP first oral feed	Infants who started oral feeding on CPAP took longer to attain **full oral feeding** (median 24 days vs 18 days) and achieved full oral feeding at a later PCA (median 37.6 weeks vs 36.6 weeks).	31% (n = 76) received **first oral feeding** on CPAP; Infants who received first oral feeding on CPAP had younger GA, lower birthweight, smaller length and head circumference than those without oral feedings on CPAP. Both infants on and off CPAP were of comparable weight and PCA at the time of first oral feeding.	No	Infants who began oral feeding on CPAP had lower GA and longer duration of intubation than infants who started oral feeding off CPAP.
**Leder 2016** [16]	**HFNC** 2-3 L/min	Not reported	Successful **initiation** of oral feeding in 17 of 50 (34%), mean CGA 35 weeks, 4 days . Remaining 34 infants (mean CGA 33 weeks, 4 days) remained nil per oral due to prematurity or medical conditions precluding oral feeding. Age differences were noted for the neonates who initiated oral feedings (greater GA, CA) however this was not statistically significant.	No	It is not the use of HFNC per se but rather patient-specific determinants of feeding readiness and underlying medical conditions that impact decisions for oral alimentation.
**Leibel 2020**	On CPAP minimum of 5cmH2O (orally fed on **LFNC** < 2 L/min**)** vs on **HFNC** minimum of 5 L/min (orally fed on 2 L/min)	Infants randomised to the HFNC group reached **full oral feeds** 7 days sooner than those randomised to CPAP. Days to full oral feeds: nCPAP 36.5 days (25.5 median); HFNC 29 days (20 median), *p* value 0.35.	Not reported	No	Feasible to perform an adequately powered RCT to confirm or refute that HFNC is associated with achieving oral feeds earlier.
**Leroue 2017** [28]	NIPPV (HFNC, CPAP, BiPAP, AVAPS); **CPAP**: 6-8cmH2O; **HFNC**: flow rate/s not reported	At time of EN initiation: 42% HHFNC, 13% CPAP, 32% bi-level support; 54% were provided with nutrition **orally**		No	EN can be provided to children on NIPPV, and in certain subsets, goal EN can be achieved while in the PICU. However, these results generate additional areas for future study about the safety and effectiveness of this practice.
**Shadman 2019** [29]	**HFNC** (flow rate/s not reported)	41% (50/123) of children treated with HFNC were **exclusively orally fed**. Compared to children who were not fed, time to discharge following HFNC completion was significantly shorter for those who were exclusively orally fed.	23% (28/123) of children treated with HFNC had mixed oral and tube feedings.	No	Children fed while receiving HFNC for bronchiolitis may have shorter time to discharge than those not fed.
**Shetty 2016** [8]	**nCPAP** (4-6cmH20) vs nCPAP then transferred to **HFNC** 2-8 L/min; No oral feeding on nCPAP, oral feeding on HFNC only.	Age to achieve **full oral feeding** was not found to be significantly different in either group. Sub-analysis of infants receiving nCPAP-only or nCPAP-then-HFNC beyond 34 weeks PMA showed that full oral feeding was achieved significantly earlier in the nCPAP-then-HFNC group (nCPAP 41 weeks PMA, 111 days of life [DOL]; nCPAP/HFNC 39.43 weeks PMA, 92 DOL).	Postnatal age at which **oral feeds first trialed** for infants requiring respiratory support after 34 weeks PMA was significantly earlier in the nCPAP/HFNC group (median PMA 34.71 weeks) vs the nCPAP group (median PMA 36.71 weeks). The nCPAP group was born at an earlier gestational age and lower birth weight.	No	In infants with BPD who required respiratory support beyond 34 weeks PMA, use of nCPAP then HFNC was associated with earlier establishment of full oral feeds.
**Shimizu 2019** [30]	**HFNC** (2 L/kg/min) vs no HFNC first oral feed	Similar ages for achievement of **full oral feeding** between the two groups 38.6 (34.4–42.3) vs 36.7 (34.6–44.4) weeks PMA respectively (*p* = 0.29). Duration from birth until the achievement of full oral feeding was earlier in the non-HFNC group than in the HFNC group (38 vs 77 median days, *p* = 0.03). The HFNC were born at a lower GA, lower BW and demonstrated more immature respiratory function than the non-HFNC group.	No significant difference in timing of **first oral feed** between the two groups: 35.3 (33.0–38.1) vs 35.5 (33.7–42.4) weeks PMA, respectively (*p* = 0.91) No difference between the two groups in duration from birth to the timings of the **first oral feed**: 52 (14–97) vs 31.5 (1–88) days, respectively (*p* = 0.07)	No	Initiation of oral feeding of VLBWIs on HFNC might be safe and might accelerate the achievement of oral feeding milestones.
**Slain 2016**	**HFNC** 2-4 L/min vs 5-6 L/min vs > 7 L/min	Children were fed in 501/794 (63%) of shifts: **434 oral,** 67 NG/ND/GT; EN was provided ‘mostly orally’ (5 children (7%) received NG or ND feeds, 3 children (4%) received GT feeds	Not reported	No	In this small patient cohort at a single institution, AEs were rare and not related to the delivered level of HFNC respiratory support. Children who were fed earlier in their PICU admission had shorted PICU stays.
**Sochet 2017** [31]	**HFNC** 4-13 L/min (0.3–1.9 L/kg/min)	97% received EN **by mouth**, 3% by NGT	Not reported	No	Oral nutrition was tolerated across a range of HFNC flow and respiratory rates, suggesting the practice of withholding nutrition in this population is unsupported.

*CPAP* continuous positive airway pressure, *HFNC* high flow nasal cannula, *OPA* oropharyngeal aspiration, *GA* gestational age, *BW* birth weight, *PMA* postmenstrual age, *PCA* post-conception age, *DOL* days of life, *VLBWI* very low birth weight infant, *CA* corrected age, *NF* nipple feeding, *TFI* total fluid intake, *OT* occupational therapist, *NC* nasal cannula, *EN* enteral nutrition, *NG* nasogastric, *ND* nasoduodenal, *GT* gastrostomy, *AE* adverse event, *SLT* speech language therapy, *LOS* length of stay, *BPD* bronchopulmonary dysplasia

Of the 16 included studies, 12 reported on full or exclusive oral feeding [8, 9, 23–26, 28–31, 33, 34]. Three studies [24, 26, 34] compared the duration to full oral feeds for infants initiating oral feeding whilst on CPAP versus those who commenced oral feeding after ceasing CPAP. Hanin [26] found that infants who initiated oral feeding while on CPAP achieved oral feeding milestones at an earlier post-menstrual age (PMA), however Dumpa [24] reported no significant difference (longer duration if started oral feeding on CPAP but achieved full oral feeding at same PMA) and LaTuga [34] reported that infants who started oral feeding on CPAP took longer to attain full oral feeding.

One study reported on oral feeding outcomes for infants on CPAP only. Bapat [23] reported on duration to full oral feeding for preterm infants with BPD who participated in a quality improvement project to enhance feeding milestones and found that infants with mild to moderate (but not for severe) BPD achieved full oral feeds earlier on their SIMPLE feeding program (median 81 days of life) vs baseline group (median 84 days of life) (*p* < 0.05).

Four studies examined oral feeding on HFNC only [9, 29–31], with one study [30] comparing duration to full oral feeds for preterm infants initiating oral feeding whilst on HFNC versus infants initiating oral feeding while not on HFNC. Shimizu [30] found that there were similar ages for achieving full oral feeding between the two groups.

Three studies [8, 25, 33] compared the duration to full oral feeding for infants supported by nCPAP versus HFNC. Glackin [25] studied infants who were orally fed on both CPAP and HFNC and reported the number of days to achieve full oral feeding was not significantly different between the two cohorts. In Shetty [8] infants were fed on HFNC only and the age to achieve full oral feeding was not found to be significantly different in either group, however a sub-analysis of infants receiving NIV beyond 34 weeks PMA showed that full oral feeding was achieved significantly earlier in the nCPAP-then-HFNC group. In Leibel [33] infants on CPAP were placed on LFNC (< 2 L/min) to orally feed and infants on HFNC had their flow reduced to 2 L/min to orally feed. Infants randomised to the HFNC group reached full oral feeds earlier.

Four studies from PICU/pediatric hospital settings [9, 28, 29, 31] reported only on the number of children receiving oral nutrition on NIV rather than duration to full oral feeds.

2) Partial oral feeding

Eight studies [8, 10, 16, 23–25, 30, 34] reported age at first oral feed. An additional four studies reported on some degree of oral feeding while receiving NIV, such as 50% of total fluid intake, mixed oral and tube feedings or small volumes via syringe.

3) Oropharyngeal aspiration, as observed on instrumental assessment

One study [14] used an instrumental swallow evaluation to assess seven infants (mean PMA 38.1 weeks) orally feeding on nCPAP. Ferrara [14] utilised VFSS to assess for aspiration during 20 swallows under two conditions: on-nCPAP (5cmH20) and off-nCPAP (1 L/min LFNC). Infants demonstrated significantly more frequent episodes of deep laryngeal penetration (43.7% vs 25.3%) and aspiration (33.5% vs 14.6%) when on-nCPAP versus off-nCPAP respectively. The remaining 15 studies did not utilise instrumental evaluation of the swallow to confirm oropharyngeal aspiration status.

#### Analysis of secondary outcomes

Secondary outcomes examined adverse events (AEs) and these were reported by 11 studies.

See Table 3 for a summary of secondary outcomes 1–3.

1. Clinical signs of oropharyngeal aspiration

**Table 3:** Summary of secondary outcomes (SO) 1, 2 and 3

Study, year	Respiratory support	SO1: Clinical signs of OPA	SO2: Aspiration pneumonia or antibiotics for suspected aspiration pneumonia	SO3: Decrease in respiratory status/signs of respiratory distress
**Bapat 2019** [23]	**CPAP** (cmH2O not specified)	Not reported	Not reported	Not reported
**Dalgleish 2016**	**CPAP** (pressure/s not reported), **HFNC** > 1.5 L/min	No cases of **suspected aspiration** based on **clinical** or radiographic **observation** and no safety concerns noted by nurses, consulting OTs or neonatologists based on individual assessment.	No cases of **suspected aspiration** based on clinical or radiographic observation.	Oral feedings were stopped at the first sign of stress, which resulted in no infant having **worsening respiratory status** or physiological instability.
**Dumpa 2020** [24]	Orally fed while on **nCPAP** 5-8cmH2O vs oral feeding after ceasing nCPAP	Not reported	Not reported	Not reported
**Ferrara 2017** [14]	**CPAP** 5cmH2O vs **LFNC** 1 L/min	Not reported	Not reported	Not reported
**Glackin 2017** [25]	**nCPAP** (pressure/s not reported) vs **HFNC** commencing at 7 L/min	Adverse events, eg. **desaturation and bradycardia**, were recorded on proforma data sheets for every feed offered. No details were provided in results- authors stated no adverse outcomes or events in any of the infants.	No **cases of aspiration** following oral feeds on nCPAP or HFNC	No **acute respiratory deterioration** occurred in any of the infants in either group.
**Hanin 2015** [26]	**nCPAP**-oral (6-8cmH2O) vs nCPAP-gavage	Frequency of physiologic and behavioral distress for all feeding sessions (*n* = 218) that resulted in termination of the bottle feeding: **Apnoea or bradycardia events** (2.7%, *n* = 6)**; Desaturation to** less than target FiO2 saturation (11%, *n* = 25); > 1 episode of **coughing** or gagging (0.4%, *n* = 1).	No clinically significant **aspiration pneumonia** No infants received any **antibiotics** during the period of nCPAP oral feeding due to suspected aspiration pneumonia.	Oral feedings were terminated when the following occurred: **increase in respiratory rate** or **work of breathing** (14%, *n* = 30). Three events (1%) required **supplemental FiO2; o**ne infant had changes in **chest x-ray.**
**Jadcherla 2016** [27]	**nCPAP** (6-8 cm H2O) vs **NC** (0.1–2.0 L/min) vs room air	Not reported	Not reported	Not reported
**La Tuga 2019**	CPAP (cmH2O not reported) vs no CPAP first oral feed	Not reported	No significant difference in **aspiration pneumonia** between infants who initiated oral feeding on CPAP (*n* = 76) compared to infants that did not begin oral feeding on CPAP (*n* = 167), with only one case of aspiration pneumonia reported in each cohort.	Not reported
**Leder 2016** [16]	HFNC 2-3 L/min	17 NICU patients had ‘successful initiation of oral alimentation’ which was defined as swallowing without overt signs of dysphagia eg. **cough** or worsening respiratory status.	Not reported	Not reported
**Leibel 2020** [30]	On CPAP minimum of 5cmH2O (orally fed on **LFO** < 2 L/min) vs on **HHHFNC** minimum of 5 L/min (orally fed on 2 L/min)	Feeding intolerance defined as “holding or decreasing the volume of feeds by the medical team due to emesis or **aspiration** (defined as **coughing** or **choking** during a feed)” nCPAP 8.33% (1/12), HHHFNC 30.77% (4/13) Aspiration vs emesis not differentiated.	None of the infants developed **aspiration** while on short-term LFO for the purpose of oral feeding.	None of the infants developed **cardio-respiratory decompensation** while on short-term LFO for the purpose of oral feeding.
**Leroue 2017** [28]	NIPPV (HFNC, CPAP, BiPAP, AVAPS); **CPAP**: 6-8cmH2O; **HFNC**: flow rate/s not reported	Not reported	Development of **pneumonia** not present at admission (*n* = 54). Difficult to discern whether a complication of feeding or natural progression of the disease.	3% (*n* = 16) of patients receiving NIPPV (*n* = 562) required **intubation** after EN initiation, 4 for elective procedures and 12 for **progressive respiratory failure.**
**Shadman 2019** [29]	**HFNC** (flow rate/s not reported)	Not reported	One fed infant had antibiotic initiation with radiological documentation of possible pneumonia and physician documentation of suspected **aspiration pneumonia.**	Not reported
**Shetty 2016** [8]	**nCPAP** (4-6cmH20) vs nCPAP then transferred to **HFNC** 2-8 L/min	Not reported	Not reported	Not reported
**Shimizu 2019** [30]	**HFNC** (2 L/kg/min) vs no HFNC first oral feed	Not reported	No clinically **significant aspiration pneumonia** in the HFNC group during oral feeding.	No **increase in oxygen requirements** between the oral feeding on HFNC vs oral feeding without HFNC groups.
**Slain 2016**	**HFNC** 2-4 L/min vs 5-6 L/min vs > 7 L/min	No documented **aspiration or choking events.** Data extracted from nursing documentation regarding 70 children who had enteral feeding (89% oral).	Not reported	Feeding-related adverse events (AEs) were categorized as **‘respiratory distress’** (*n* = 9) or ‘emesis’ (*n* = 20). AEs documented in 29 of 501 (6%) nursing shifts (434 shifts with oral feeds).
**Sochet 2017** [31]	**HFNC** 4-13 L/min (0.3–1.9 L/kg/min)	Not reported	Development of **aspiration-related respiratory failure** occurred in 1 (0.8%) patient	Interruptions in enteral nutrition occurred in 12 (9.1%) children, 10 for **tachypnoea**, 1 for **increased work of breathing**

*OPA* oropharyngeal aspiration, *BPD* bronchopulmonary dysplasia, *CPAP* continuous positive airway pressure, *HFNC* high flow nasal cannula, *HHHFNC* heated and humidified high flow nasal cannula, *LFNC* low flow nasal cannula, *LFO* low flow oxygen, *NC* nasal cannula, *NIPPV* noninvasive positive-pressure ventilation, *BiPAP* Bilevel positive airway pressure, *AVAPS* average volume-assured pressure support

Clinical signs suggestive of oropharyngeal aspiration (OPA) include coughing, choking, noisy or wet breathing, wet vocalisations, wheeze, recurrent pneumonia, gagging, congestion, tachypnoea, bradycardia, apnoea, colour changes, oxygen desaturations and voice changes during and/or after feeds [36, 37]. Six studies [9, 10, 16, 25, 26, 33] reported on clinical signs of aspiration, including the incidence of specific clinical signs or general statements.

2) Aspiration pneumonia or use of antibiotics for clinically suspected aspiration pneumonia

Nine studies [10, 25, 26, 28–31, 33, 34] reported on the incidence of aspiration pneumonia and/or use of antibiotics for clinically suspected aspiration pneumonia.

3) Decrease in respiratory status/respiratory distress

Eight studies [9, 10, 25, 26, 28, 30, 31, 33] reported on the incidence of deterioration in respiratory status or respiratory distress in response to oral feeding on nCPAP or HFNC.

4) Other secondary outcomes

Two studies reported on behavioural responses during oral feeding on nCPAP or HFNC. Hanin [26] reported on the frequency of oral feedings on nCPAP that were ceased in response to ‘more than one episode of coughing or *gagging*’ (0.4%, *n* = 1, total oral feedings = 218), however the descriptors were nonspecific/unclear if coughing or gagging. Dalgleish [10] reported that no infant exhibited symptoms of ‘*ongoing or recurring distress related to nipple feeding opportunities’*.

Two studies [23, 29] reported on readmissions and one study [31] reported death as an outcome, stating zero mortality for children with acute viral bronchiolitis on HFNC receiving enteral nutrition. No studies reported on oral aversion/feeding refusal or gastroesophageal reflux.

### Additional analysis

A meta-analysis was not possible due to significant heterogeneity between included studies.

## Discussion

This study aimed to systematically review the literature to evaluate whether oral feeding on nCPAP and HFNC facilitates transition to full oral feeds without adverse effects. The findings are insufficient to conclude whether commencing oral feeding whilst on nCPAP or HFNC facilitates transition to full oral feeding and the risk of adverse events including oropharyngeal aspiration is unclear.

Duration to full oral feeding for participants on nCPAP or HFNC was associated with gestational age (GA) at birth and severity of respiratory disease, which also reflects literature for children without NIV support. The development of oral feeding skills and duration to full oral feeding are known to be related to GA at birth for preterm children, with extremely and very preterm infants achieving full suck feeds later [38, 39]. Infants and children with respiratory disease are also at increased risk of feeding difficulties, without the added complication of NIV [40]. Reduced suck-swallow-breathe coordination, poor feeding efficiency and endurance, weak sucking pressures and difficulties ingesting adequate intake have been reported in this population [41, 42]. Studies matching infants requiring NIV with those no longer requiring NIV are likely comparing cohorts with different respiratory disease severity and therefore feeding outcomes will likely be different, regardless of the use of NIV.

Reported adverse events (AEs) due to oral feeding on NIV varied. AEs during oral feeding may include physiological, respiratory and behavioural responses including desaturation, bradycardia, increase in work of breathing or coughing/choking. Information regarding AEs were mostly obtained retrospectively via chart review or a lack of documented problems was reported, eg. ‘*no documented aspirations or choking events*’ [9] presenting opportunities for missed data. The majority of studies did not specify tools used to record AEs or staff training in recognising AEs, therefore reported rates of AEs may be low.

Only a small number of studies reported on the use of supportive feeding practices, including assessing feeding readiness, reading infant cues, stopping a feed at the first sign of stress or physiologic instability, use of modified teat flow rate, feeding the infant in a sidelying position and use of external pacing. Oral feeding strategies are beneficial to support physiological stability and reduce the risk of cardiorespiratory events and infant stress/disengagement during oral feedings in preterm or unwell infants [32]. Lack of implementation of supportive strategies may contribute to AEs and increase risk of aspiration during oral feeding on NIV.

This review identified a lack of studies utilising instrumental assessment tools for assessing swallow safety. While VFSS and FEES are considered gold standards for evaluating aspiration, only one study [14] used VFSS to determine aspiration status of infants orally feeding while on nCPAP. Based on their preliminary findings of increased laryngeal penetration and aspiration in children on nCPAP, Ferrera and colleagues reported that their ethics committee discontinued the trial, and their institutional practice was changed to have children placed NPO whilst receiving nCPAP support. No studies to date have utilitised VFSS or FEES to assess aspiration status of infants and children orally feeding while on HFNC. Most studies reported on clinical signs of aspiration only. Signs and symptoms of aspiration are known to be age dependent, with children experiencing high rates of silent aspiration, and clinical evaluation having lower sensitivity in detecting aspiration [36, 37] thus likely to underreport the true incidence of aspiration. In addition, premature infants and previously healthy infants with RSV bronchiolitis are known to be at increased risk of aspiration [40, 43] in the absence of NIV, so it can be difficult to determine if the clinical signs of aspiration are related to the underlying condition or to the presence of NIV. Some studies reported on the use of antibiotics for clinically suspected aspiration pneumonia or use of chest x-ray to assess for aspiration, which has poor sensitivity as a diagnostic tool for microaspiration [44]. The clinical response to aspiration can depend on the frequency of aspiration, volume and type of material aspirated and health status of the patient [45]. Undetected aspiration may prolong respiratory support requirements and lead to negative outcomes such as oral aversion and respiratory morbidities.

In addition, studies varied in their definition of HFNC. A 2014 Cochrane review stated that ‘high flow’ has not been well described in the literature, and defined HFNC in children as having flow rates of ≥ 2 L/min [46]. During HFNC therapy, mean nasopharyngeal pressure increases as flow increases but decreases with infant weight [7, 12]. Flow rates can therefore have different impacts on children depending on their size, so HFNC is best described as a weight-adjusted flow rate (ie. L/min/kg). Use of this unit would allow more accurate comparison between cohorts and to determine if there is a correlation between flow rate and adverse events.

Finally, another factor of consideration with HFNC is the effect of mouth position on oral and pharyngeal pressures. Wilkinson et al. [7] demonstrated that mouth position during HFNC had little effect on pharyngeal pressures, likely due to nasal leak, however Kubicka et al. [47] reported that the amount of pressure generated during HFNC was related to the degree of mouth opening. A sealed oral cavity during suck feeding therefore may have the potential to further increase pharyngeal pressures and impact swallow safety, however this may be more variable for older children and the type of utensils being used for eating and drinking.

Limitations of this review include a small number of studies retrieved from our search, small sample sizes in some included studies, the retrospective nature of many studies and only one study utilising instrumental assessment of the swallow to determine aspiration status (on nCPAP). Given the lack of clear determination of aspiration status when orally feeding whilst receiving nCPAP or HFNC, it is difficult to provide clear guidance as to what should be best practice clinical care for infants and children. There is a clear need for future prospective research of high quality cohort or controlled trials to determine aspiration status using instrumental evaluation. Additionally, future research should evaluate the range of clinical practices that are provided to infants and children of different ages and with different underlying respiratory conditions. Future studies should include a comparison of both and utilizing instrumental evaluation such as FEES or VFSS to determine aspiration, as well as duration on respiratory support and length of stay. Older infants and children (often with an underlying respiratory infectious disease) receiving nCPAP or HFNC may be changed to low flow supplemental oxygen for oral feeding (due to concerns about aspiration risk) and returned to their high flow/pressure respiratory support after the feed/mealtime. We have no way of knowing whether this practice supports or negatively impacts on aspiration risk for these children. Others may be fed, or eat and drink, whilst on their respiratory support for mealtimes. These scenarios are commonly encountered although there is no strong evidence utilizing carefully designed prospective studies with instrumental evaluation to determine aspiration and reflecting these practices to support clinical guidelines. We recommend caution if orally feeding on CPAP or HFNC due to the potential for oropharyngeal aspiration and that each child should have a clinical feeding evaluation by a trained dysphagia therapist with the opportunity for VFSS or FEES to support clinical care, until further strong evidence is available.

## Conclusion

This systematic review examined oral feeding efficacy and safety for infants and children receiving nCPAP and HFNC. Variations in NIV definitions, small cohort numbers, a wide variety of study outcomes and poorly defined AEs impacted on the ability to conduct a meta-analysis. Findings are insufficient to conclude whether commencing oral feeding whilst on nCPAP or HFNC facilitates transition to full oral feeding without adverse events. Further research is warranted, including prospective studies with instrumental assessment of swallow safety, in particular on HFNC for which instrumental assessment has not yet been utilitied. This will assist in the future development of clinical guidelines and recommendations for best practice with these populations.

## Data Availability

All data generated or analysed during this study are included in this published article (and its supplementary information files).

## Abbreviations

nCPAP: Nasal continuous positive airway pressure
HFNC: High flow nasal cannula
LFNC: Low flow nasal cannula
NIV: Non-invasive ventilation
NICU: Neonatal intensive care unit
PICU: Pediatric intensive care unit
CNLD: Chronic neonatal lung disease
BPD: Bronchopulmonary dysplasia
AE: Adverse event
PMA: Post-menstrual age
GA: Gestational age
OPA: Oropharyngeal aspiration
VFSS: Videofluoroscopic swallow study
FEES: Fiberoptic endoscopic evaluation of swallowing
RCT: Randomised control trial
NPO: Nil per oral
